# α‐Dioxygenases (α‐DOXs): Promising Biocatalysts for the Environmentally Friendly Production of Aroma Compounds

**DOI:** 10.1002/cbic.202100693

**Published:** 2022-02-15

**Authors:** In Jung Kim, Thomas Bayer, Henrik Terholsen, Uwe T. Bornscheuer

**Affiliations:** ^1^ Institute of Biochemistry, University of Greifswald Felix-Hausdorff-Str. 4 17489 Greifswald Germany

**Keywords:** alpha-dioxygenase, aroma compounds, cyanobacteria, fatty acids, fatty aldehydes, (high-throughput) aldehyde detection

## Abstract

Fatty aldehydes (FALs) can be derived from fatty acids (FAs) and related compounds and are frequently used as flavors and fragrances. Although chemical methods have been conventionally used, their selective biotechnological production aiming at more efficient and eco‐friendly synthetic routes is in demand. α‐Dioxygenases (α‐DOXs) are heme‐dependent oxidative enzymes biologically involved in the initial step of plant FA α‐oxidation during which molecular oxygen is incorporated into the C_α_‐position of a FA (C_n_) to generate the intermediate FA hydroperoxide, which is subsequently converted into the shortened corresponding FAL (C_n‐1_). α‐DOXs are promising biocatalysts for the flavor and fragrance industries, they do not require NAD(P)H as cofactors or redox partner proteins, and they have a broad substrate scope. Here, we highlight recent advances in the biocatalytic utilization of α‐DOXs with emphasis on newly discovered cyanobacterial α‐DOXs as well as analytical methods to measure α‐DOX activity *in vitro* and *in vivo*.

## Graphical abstract

**α‐Dioxygenases (α‐DOXs)** are promising biocatalysts that catalyze the oxidation of fatty acids to produce (one carbon‐shortened) fatty aldehydes, which are used as flavors and fragrances. This comprehensive review summarizes the recent advances in the biotechnological utilization of α‐DOXs with focus on newly discovered cyanobacterial α‐DOXs together with analytical methods to measure α‐DOX activity.

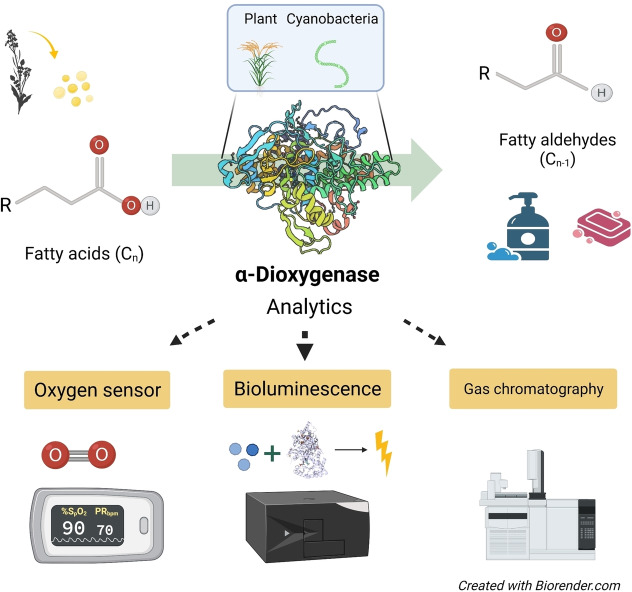

## 1 Introduction

Aldehydes, identified first by the German chemist Justus von Liebig in 1835, are highly reactive and industrially relevant compounds.^[1]^ Aliphatic fatty aldehydes (FALs), typically derived from fatty acids (FAs), possess structural diversity depending on the carbon chain length and the existence/number/position of double bonds or other substituents, which translates into different physicochemical properties.^[2]^ FALs have a high commercial value as fragrances and flavor agents with a market‐value of 35 billion USD.^[3]^ The medium‐chained octanal, nonanal, and decanal are noted as green‐floral and citrus flavors, and considered as suitable scents in perfumes.^[1a]^ Consequently, various aliphatic aldehydes are formulated in the mixture of many renowned perfumes.^[3]^ Unsaturated aldehydes like (*E*)‐2‐hexenal and (*Z*)‐11‐hexadecanal are produced by insects and are good candidates for pheromone synthesis.^[4]^ In addition, methyl‐branched aldehydes (*e. g*., 12‐methyltridecanal) are used as food supplements, adding a meaty flavor.[^2^, ^5^]

Although widely distributed in nature, most FALs like many other aldehydes do not accumulate in microorganisms due to their cytotoxicity and high reactivity, resulting in their rapid conversion into the corresponding alcohols.^[6]^ Hence, most aldehydes utilized in today's industries are chemically produced from petroleum. As an alternative, utilization of FAs obtained from plant oils as a renewable and sustainable feedstock is currently increasing.^[7]^ For this process, a two‐step reaction is typically performed due to the high reactivity of aldehydes; this involves the initial reduction of the carboxylic acid to an alcohol and the subsequent re‐oxidization to the aldehyde.^[8]^ In addition, the state‐of‐the art technology is based on Mn‐ or Ni‐catalyzed selective reduction of a carboxylic acid to an aldehyde.^[9]^ Alternatively, the aldehyde is directly synthesized from the corresponding primary alcohol using (toxic) oxidants like chromium VI.^[10]^ A much less toxic catalyst, TEMPO (2,2,6,6‐tetramethylpiperidinyloxyl), can be used for the oxidization of alcohols to aldehydes using O_2_ as the oxidant, but this is encountered with lower selectivity.^[11]^ Overall, chemical synthesis of aldehydes requires high energy consumption and intensive labor.[^1a^, ^12^] Driven by the call from consumers for “natural” products, preferably starting from renewable resources through more sustainable processes, companies producing foods/beverages, perfumes, cleansing products, and cosmetics call for alternatives to traditional extractive^[13]^ and chemical methods towards the synthesis of aliphatic aldehydes. These include the utilization of microbial or enzyme‐mediated bioprocesses, which are considered environmentally‐friendly and natural. Recently, the following FAL‐yielding biosynthetic enzymes have been employed: fatty acid acyl‐CoA/acyl carrier protein (ACP) reductases (FARs),^[14]^ carboxylic acid reductases (CARs),^[15]^ alcohol dehydrogenases (ADHs),[^15c^, ^16^] alcohol oxidases (AOXs),^[16]^ and α‐dioxygenases (α‐DOXs).[^2^, ^15b^, ^18^] Among them, α‐DOXs, which are heme‐dependent and act on FAs (C_n_) yielding the corresponding FALs (C_n‐1_) and CO_2_,[^17a^, ^18^] have several advantages including no requirement of cofactors, such as NAD(P)H or redox partner enzymes. Furthermore, they are capable of synthesizing odd‐numbered products.[^2^, ^15b^, ^18a^, ^18b^, ^19^] However, a comprehensive review of α‐DOX as biocatalyst is lacking. Since the biological function and enzymatic mechanism of α‐DOXs have been substantially reviewed elsewhere,^[20]^ this review focuses on the recent advances of α‐DOX research from a biotechnological perspective, highlighting newly discovered cyanobacterial α‐DOXs, and analytical methods to measure α‐DOX's activity *in vitro* and *in vivo*.

## 2 Biosynthetic Enzymes Yielding Fatty Aldehydes

**Figure 1:**
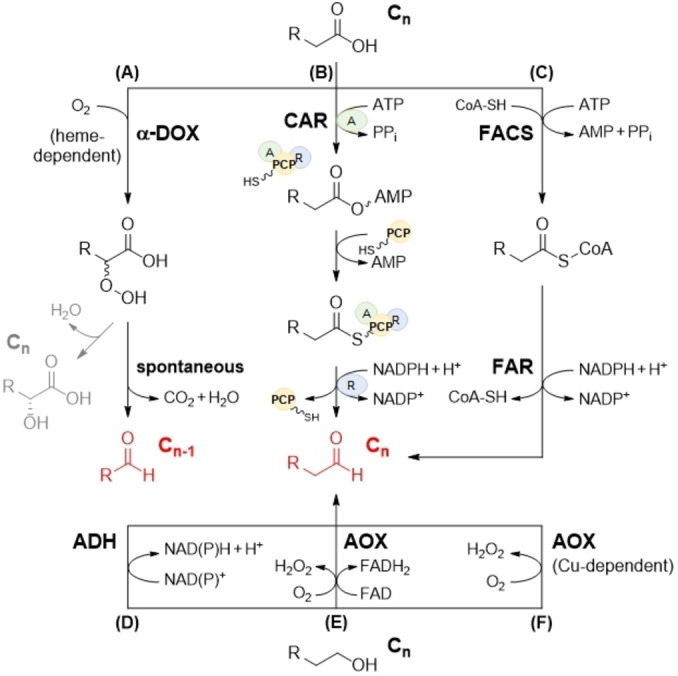
Enzymes for the synthesis of fatty aldehydes (FALs). Carboxylic acids (FFAs; top) and primary alcohols (fatty alcohols; bottom) serve as substrates for the biocatalytic production of FALs (red). (A) Heme‐dependent α‐DOXs accept FFAs (C_n_) and utilize O_2_, yielding the corresponding 2‐hydroperoxide FA intermediates, which can react to produce (*R*)‐2‐hydroxy FAs (C_n_; grey) and/or be spontaneously decarboxylated, yielding the desired FALs (C_n‐1_).^[28]^ (B) CARs consist of three domains: an adenylation domain (A; green), a peptidyl carrier protein (PCP; yellow) containing a 4′‐phosphopantetheine arm, and a reduction domain (R; blue). The thermodynamically stable carboxylic acids are reduced by CAR at the expense of ATP and NADPH yielding FALs (C_n_);^[23b]^ the involved domains are highlighted throughout the reaction scheme. (C) FARs cannot convert FFAs but AFAs, which are synthesized by the activity of a fatty‐acyl‐CoA synthase (FACS) and are subsequently reduced to FALs (C_n_) by NADPH‐dependent FARs.^[18c]^ (D) ADHs can oxidize fatty alcohols depending on different nicotinamide cofactors but also FAD‐dependent ADHs like AlkJ have been reported (not shown).^[15c]^ AOXs use O_2_ and depend either on (E) FAD or (F) Cu ions as the cofactor to oxidize primary alcohols to the corresponding FALs (C_n_).^[17a]^

Most commonly, the biosynthetic pathways for FALs have utilized FARs[^14^, ^21^] or CARs[^15a^, ^15b^, ^15d^, ^15e^] (Figure 1, Table 1). FARs accept activated FA (AFA) precursors, typically CoA‐derivatives, as substrates and are often characterized by low activity and the need for the use of whole‐cell systems to ensure their activation through CoA, rendering them of minor significance for industrial (large‐scale) productions (Figure 1C). Contrary, the utilization of free FAs (FFAs) has advantages since FFAs are readily available from cheap and renewable biomass.^[22]^ CARs not only accept FFAs as substrates; many CAR enzymes exhibit broad substrate scope (Figure 1B).[^15a^, ^15b^, ^15d^, ^23^] However the reduction of carboxylates is demanding, and CARs require adenosine triphosphate (ATP), Mg^II^, and reduced nicotinamide adenine dinucleotide phosphate (NADPH). Additionally, the activity of a co‐expressed phosphopantetheinyl transferase (PPTase) converts apoCARs into holoCARs. Both, the cofactor dependence and posttranslational modification make *in vitro* applications less feasible.[^15a^, ^15b^, ^15d^] Consequently, CARs as well as FARs have been operated in whole‐cells such as *Escherichia coli*, *Saccharomyces cerevisiae*, or *Acinetobacter baylyi*.[^14^, ^21^, ^23b^] Cofactors (*e. g*., NADPH and ATP) and acetyl‐CoA as well as posttranslational modifications are efficiently supplied and recycled by the host metabolism and performed in the same cell.^[24]^ Complementary to enzymatic reductions of FAs, FALs can be obtained through the oxidation of the corresponding aliphatic alcohols by oxidoreductases such as ADHs and AOXs (Figures 1D–F), although only a few types have been investigated in whole‐cell systems for this purpose.^[6]^ AlkJ, for example, a flavin adenine dinucleotide (FAD)‐dependent ADH from *Pseudomonas putida* has been successfully employed in the oxidation of structurally diverse primary alcohols. Due to the cofactor requirement and the association of AlkJ with the membrane, target transformations were carried out in living cells exclusively.^[15c]^ Furthermore, FAD‐dependent AOXs irreversibly oxidize alcohols to the corresponding aldehydes in the presence of molecular oxygen (O_2_). Hydrogen peroxide (H_2_O_2_) is formed as byproduct and further converted into O_2_ and water by catalases *in vitro* and *in vivo*.[^18c^, ^24b^, ^25^] Recently, Heath *et al*. engineered a choline oxidase by structure‐guided directed evolution towards the oxidation of hexanol into its corresponding medium‐chain aldehyde.

**Table 1:** Comparison of fatty aldehyde synthesis in whole‐cell systems using various enzymes.^[a]^

	FAR				CAR		α‐DOX						
Host	*E. coli*		*S. cerevisiae*		*E. coli*		*E. coli*				*S. cerevisiae*		
Enzyme name	*Se*FAR		*Ma*FAR		*Mm*CAR		*Cal*DOX *Lep*DOX	*Os*DOX	*Cal*DOX *Lep*DOX	*Os*DOX	*Os*DOX		
FA substrate	AFA				C8:0	12 : 0	C10 : 0	C12 : 0	C14 : 0	C16 : 0	C14 : 0	C16 : 0	C18 : 0
Substrate loading [mm]	ND^[b]^				100	30	5	30	5	5	∼0.04		
FAL product	C16 : 0	C18 : 0	C16 : 0	C18 : 0	C8 : 0	12 : 0	C9:0	C11:0	C13:0	C15:0	C13 : 0	C15 : 0	C17 : 0
Yield [mm]	0.146	0.075	0.004	0.004	96	0.7	5 0.43	10	5 2.5	4	0.003	0.010	0.002
Conversion [%]	ND^[b]^				96	2.3	100 8.6	67	100 50	80	7.5	25	5
Time [h]	Not reported		48		27	51	0.33 1	27	0.66 1	2	48		
Cofactor	NADPH				ATP NADPH Mg^2+^		Heme						
References	[14c]		[21b]		[15e]	[15b]	[18b]	[15b]	[18b]	[18a]	[41]		

[a] There are no reports for whole‐cell biotransformations using ADH and AOX for fatty aldehyde synthesis. [b] ND, not determined.

Whereas the activity for the desired oxidation was satisfyingly increased *in vitro*, it significantly dropped for alcohols beyond heptanal.^[17b]^ A similar substrate scope could be determined for an AOXs from the phytopathogenic fungi *Colletotrichum graminicola* (*Cgr*AOX).[^26^, ^27^] *Cgr*AOX is a member of the galactose 6‐oxidase/glyoxal oxidase family of mononuclear copper‐radical oxidases referred to as Auxiliary Activity Family 5 (AA5) and it readily oxidizes short‐ to medium‐chain alcohols (C_2_‐C_7_). In contrast to AlkJ or FAD‐dependent AOXs, the oxidation of alcohols to the corresponding aldehydes by *Cgr*AOX utilizes Cu^I^ and O_2_, which are oxidized to Cu^II^ and reduced to H_2_O_2_, respectively.[^26^, ^27^]

More recently, α‐DOXs complemented the set of biocatalysts for the manufacturing of flavor and fragrance aldehydes (Table 1).[^2^, ^15b^, ^18a^, ^18b^] Some advantages relevant for future industrial applications were introduced earlier in this review but also include the capability of α‐DOXs to directly convert (non‐activated) FFA that can be supplied exogenously. Unlike CAR, neither additional cofactors nor the co‐expression of accessory enzymes are required; α‐DOXs only require atmospheric O_2_ for their catalysis (Figure 1A).[^20^, ^28^] From a biotechnological point of view, the most distinctive characteristic of α‐DOXs is the possible production of rare, odd‐numbered FALs from abundant even‐chained FA substrates. Therefore, α‐DOXs will be reviewed in detail in the following sections.

## 3 Untapped Potential: α‐DOX as Biocatalyst

### 3.1 Biological role and chemical mechanism

In nature, α‐DOXs are widespread among the plant kingdom. They participate in the physiologically important α‐oxidation of long chain FAs (LCFAs)^[20]^ yielding oxylipin, for example, which is of great biological significance in plants and involved in signaling pathways, wound healing, or the defense against bacteria.^[29]^ The physiological role of α‐DOX enzymes in plants is substantiated by their upregulated expression in response to various biotic and abiotic stresses like pathogen and herbivore infection, oxidative damage, drought, or heavy metals.[^29b^, ^30^]

**Figure 2:**
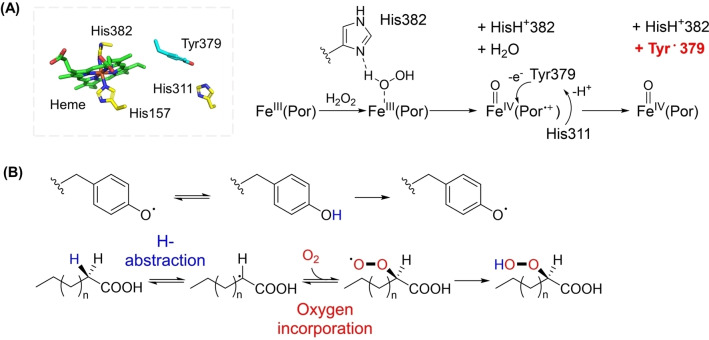
(A) α‐DOX‐mediated catalysis is initiated by the radical of a conserved tyrosine, which is generated by the activated heme moiety independently of the FA substrate. (B) The catalytic tyrosine facilitates FA oxidation through H‐abstraction from the C_α_‐H bond of a FA, to which an oxygen molecule is inserted, resulting in the (*R*)‐2‐hydroperoxide. This scheme was created based on the structure of *Os*DOX (PDB code: 4kvk).

In α‐DOX‐catalyzed reactions, α‐DOXs incorporate O_2_ into the C_α_‐position of FFAs in a regio‐ and stereoselective manner, forming the (*R*)‐2‐hydroperoxide (Figure 1 and Figure 2).^[31]^ The first step is initiated in the α‐DOX family by a radical of the catalytic tyrosine (Tyr⋅), which is strictly conserved and stabilized by π‐stacking with adjacent phenylalanines.^[28]^ The tyrosyl radical is independently generated prior to FA oxidation during which the heme moiety is oxidized by H_2_O_2_ as the radical initiator, converting ferric protoporphyrin IX, Fe^III^(Por), to ferryl Fe^IV^=O(Por). Then, FA oxidation occurs by hydrogen transfer between Tyr⋅ and the H‐bond in the C_α_‐position of the FA. Mediated by Tyr⋅, FA oxidation initially undergoes C_α_‐H homolysis followed by O_2_ radical trapping to the C_α_‐position, eventually producing the (*R*)‐2‐hydroperoxide of the FA. The unstable hydroperoxy FA (C_n_) is finally converted into the one carbon‐shortened FAL (C_n‐1_), by the spontaneous (*i. e*., non‐enzymatic) release of CO_2_ and H_2_O. On this mechanistic ground, the heme is considered as an indispensable prosthetic group for the catalytic machinery of α‐DOX.^[28]^

### 3.2 Non‐plant origin α‐DOXs

To date, most studies have been done with plant α‐DOXs.[^18a^, ^29b^, ^32^] Their origins range from primitive moss to higher flowering plants.[^18a^, ^29b^, ^32^] Among them, *Os*DOX from *Oryza sativa* and *At*DOX from *Arabidopsis thaliana* have been systematically investigated with respect to their biochemical mechanism,[^28^, ^31^] molecular functions,^[33]^ and crystal structures.^[34]^ Accordingly, this knowledge serves as useful basis for identifying α‐DOX homologues. In addition to land plants, the potential occurrence of α‐DOXs in green algae was suggested for the first time in 1999 by detecting the (*R*)‐2‐FA hydroperoxide product after incubation of FA with crude enzyme isolated from *Ulva pertusa*.^[35]^ Very recently, three cyanobacterial α‐DOXs (*Cs*DOX from *Crocosphaera subtropica*, *Cal*DOX from *Calothrix parietina*, and *Lep*DOX from *Leptolyngbya* sp.) were identified and characterized.[^2^, ^18b^] This is a significant contribution to the field as *Cs*DOX, *Cal*DOX, and *Lep*DOX are the first prokaryotic α‐DOXs identified so far. With the aid of sequence information available in public databases, an unexpected wide distribution of (putative) α‐DOX‐coding sequences across taxonomy (*e. g*., prokaryotes, fungi, and metazoa) could be revealed.^[18b]^ Cyanobacterial α‐DOXs are phylogenetically distinct from plant α‐DOXs with a low sequence identity of around 40 %; however, they share the conserved active site Tyr and two histidine residues as heme ligands, which are essential for α‐DOXs’ oxidative catalysis (Figure 2). Cyanobacterial α‐DOXs were experimentally proven to be heme proteins by observing Soret peak intensities that correlated with activities^[18b]^ and observed to produce FALs (C_n‐1_) from various FAs (C_n_).^[2]^ The substrates investigated included rare FAs like methyl‐branched FAs, the common even‐numbered saturated FAs (SFAs) as well as several unsaturated FAs (USFAs) (Table 1).[^2^, ^18b^]

In spite of the equivalent catalytic residues, substrate spectra between plant and cyanobacterial α‐DOXs are different (Table 2).[^2^, ^18b^, ^33a^] Whereas plant α‐DOXs well‐accepted longer SFAs (*e. g*., C12:0<C14:0<C16:0), cyanobacterial α‐DOXs studied up to date show preference for shorter SFAs (*e. g*., C12:0>C14:0>C16:0). Interestingly, *Cal*DOX even accepted the shorter C10:0 as the best substrate, which was not the case for *Lep*DOX and *Cs*DOX.

**Table 2:** Comparison of molecular properties of α‐DOX from plants and cyanobacteria.

		Plants		Cyanobacteria		
		*Os*DOX	*At*DOX	*Cs*DOX	*Cal*DOX	*Lep*DOX
Origin		*Oryza sativa*	*Arabidopsis thaliana*	*Crocosphaera subtropica*	*Calothrix parietina*	*Leptolyngbya* sp.
Oligomeric state (# of subunits)		ND^[a]^	Varying (1, 4, 6, 8, 10)	ND^[a]^	Heterogeneous (1 & undefined aggregate)	
Optimal conditions		ND^[a]^	pH 7.5 ‐ 8	pH 7.5 50 mM NaCl 25 °C	pH 6–9 30 to 35 °C	pH 6–9
Substrate scope	Investigated FAs	Even‐chain SFAs (C6–C20) Even‐chain USFAs (C16–C20 with varying unsaturated bonds) Modified FAs (C18)		Even‐chain SFAs (C6–C16) Methyl‐branched SFAs (C12–C15)	Even‐chain SFAs (C6–C18) Even‐chain USFAs (C16–C18 with varying unsaturated bonds)	
					
	Preferred FAs^[b]^	C14 : 0 C16 : 0 C16 : 1(9*Z*) C18 : 1(9*Z*) C18 : 2(9*Z*,12*Z*) C18 : 2(9*E*,12*E*) C18 : 3(9*Z*,12*Z*,15*Z*) C20 : 1(11*Z*) C20 : 3(8*Z*,11*Z*,14*Z*)	C18 : 1(9*Z*) C18 : 2(9*Z*,12*Z*) C18 : 3(9*Z*,12*Z*,15*Z*)	C12 : 0 C14 : 0	C10 : 0 C12 : 0 C14 : 0 C16 : 0	C12 : 0 C14 : 0 C16 : 0
References		[33a]	[32a, 33a]	[2]	[18b]	

[a] ND, not determined. [b] Substrates with relative activities of >75 % were defined as preferred FAs in this review.

Given that FALs with a length of C_8_ to C_13_ are frequently used as fragrances and flavorings, the activity of cyanobacterial α‐DOX towards shorter‐chained FAs might be industrially relevant. Homology modeling suggested substrate‐recognition residues, interacting with C_8_ and C_12_ of FAs that potentially determine the substrate scope of members of the α‐DOX family^[18b]^ and opens up new lines of research in the future.

### 3.3 Applications of α‐DOX in whole‐cell systems

#### 3.3.1 Synthesizing FALs as the final product

For functional and structural characterization, enzymes need to be isolated and purified in sufficient amounts. However, plant α‐DOXs, natively localized in lipid droplets, are known to associate with membranes through amphipathic N‐terminal helices.[^32b^, ^34a^] This contributes to the low functional yields of recombinant soluble α‐DOX protein expressed in heterologous hosts like *E. coli*. Hence, elaborate extraction and purification protocols involving detergents have to be used prior to characterization of (novel) α‐DOXs.[^2^, ^34a^] Noteworthy, detergents can crucially affect the oligomeric state of α‐DOX. The oligomeric states of α‐DOXs were reported to highly vary or be heterogeneous depending on the detergent system, which could be closely associated with the catalytic activity of the enzymes[^18b^, ^32a^] (Table 2). This indicates that an additional purification step based on size‐exclusion chromatography, for example, may be required to obtain homogeneous and functional proteins. Collectively, the intrinsic properties of α‐DOX related to its solubility make them industrially incompatible as high cost is posed for biocatalyst production. Consequently, α‐DOXs are best suited for applications in whole‐cell systems (with intact membranes) rather than applying isolated and purified enzymes.[^15b^, ^18a^, ^18b^] α‐DOXs have been operated in resting or growing *E. coli* cells; *Os*DOX, for example, successfully converted a series of supplemented SFAs (C_8_ to C_16_) into the corresponding products.[^15b^, ^18a^] According to a study directly comparing the performance of *Os*DOX and CAR from *Mycobacterium marinum* (*Mm*CAR) in *E. coli* growing cells with minimal M9 media, *Os*DOX was superior to *Mm*CAR when producing FALs although the carbon length of the products differed due to the different mechanisms of two enzymes.^[15b]^

Specifically, in contrast to *Mm*CAR‐catalyzed reaction that produced only 0.7 mm dodecanal (C_12_) after 51 h from 30 mm dodecanoic acid (C12:0), the maximal concentration of undecanal (C_11_) produced by *Os*DOX‐containing growing cells was 10 mm after 27 h. The higher performance of *Os*DOX was still valid even when the product concentrations were normalized by cell density. The authors demonstrated that this result could be due to the dominant production of fatty alcohol from the reduction of FALs by *Mm*CAR‐expressing cells.^[15b]^ Although a biphasic system using *n*‐decane ‐ introduced in an effort to circumvent the FAL reduction ‐ improved the yield of FAL and dramatically reduced the proportion of alcohol in *Os*DOX‐expressing cells, it was not very effective in CAR‐expressing cells.^[15b]^ In another study by Kaehne *et al*., *Os*DOX in resting cells resulted in the production of about 4 mm pentadecanal (C_15_) from 5 mm hexadecanoic acid (C16:0) after 2 h reaction time. After preincubation of cells with Triton X‐100 as the detergent, full conversion was achieved within only 1 h.^[18a]^ This result indicates that a detergent can improve the product yield of α‐DOX‐mediated biotransformations possibly by facilitating substrate uptake through cell permeabilization and/or by increasing solubility of hydrophobic substrates. As introduced above, the newly discovered cyanobacterial α‐DOXs (*e. g*., *Cal*DOX and *Lep*DOX), were shown to function well in *E. coli* and they successfully converted exogenously supplemented decanoic acid (C10:0) and tetradecanoic acid (C14:0), following previous experimental set‐ups employing *Os*DOX.^[18b]^ Particularly, *Cal*DOX displayed a better performance than *Os*DOX, reaching full conversion at 5 mm initial substrate load for both substrates within 40 min without a preincubation with a detergent (Table 1).

However, one drawback of whole‐cell biocatalysts can be the formation of side‐products. From FALs, mainly fatty alcohols are produced *in vivo*, a challenge that has been well‐addressed in various studies.[^6^, ^15b^, ^36^] The further reduction of overproduced FALs to fatty alcohols is caused by endogenous aldo‐keto reductases (AKRs) and ADHs both acting in the detoxification of reactive aldehydes.[^6^, ^36a^] However, *E. coli* cells expressing α‐DOXs displayed reduced or no detectable alcohol formation and this might be attributed to different cell physiologies associated with the redox state of cells, for example.[^15b^, ^18b^] Besides fatty alcohols, hydroxyl FAs (C_n_) can be formed as side‐products due to the peroxidase activities of many α‐DOXs (Figure 1);[^32b^, ^37^] however, *Os*DOX does not exhibit peroxidase activity,^[33b]^ qualifying it as a good biocatalyst.

#### 3.3.2 Synthesis of value‐added products from FAL intermediates

**Figure 3:**
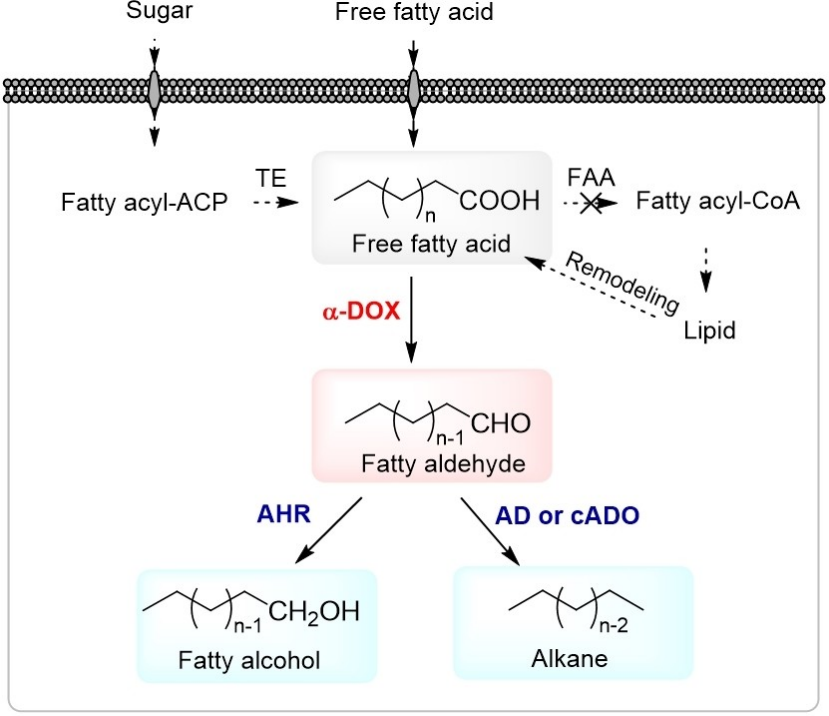
Schematic diagram for the α‐DOX‐mediated production of FALs, fatty alcohols, and alkanes in a microbial system from FA. The whole cell catalysis utilized an α‐DOX for FAL synthesis as the final product[^15b^, ^18a^] and subsequent pathways were constructed by introducing AHR^[19]^ and cADO (or AD)[^19^, ^41^] to convert the FAL intermediates into fatty alcohols and alkanes, respectively. Free FAs can be obtained by either *de novo* synthesis from simple sugars (dashed line) or exogenous supplementation. Abbreviations: AHR; aldehyde reductase, AD; aldehyde decarbonylase, cADO; cyanobacterial aldehyde deformylating oxygenase, TE; thioesterase, FAA; acyl‐CoA synthetase.

As already stated above, α‐DOXs can produce highly demanded FALs from FFAs. Further, FALs can be used as intermediates towards other value‐added products (Figure 3). Although fatty alcohols are undesirable when FALs are the desired final products, they have applications as fuels, fragrances, detergents, and surfactants.[^15a^, ^19^, ^38^] Numerous studies targeted the fatty alcohol biosynthesis directly from activated FAs by FARs, or from FAL by aldehyde reductases and related enzymes in whole‐cell systems.[^15a^, ^19^, ^21b^, ^39^] Using these biocatalysts, medium‐ to long‐chain fatty alcohols were obtained with titers of 0.06 g/l ^[39b]^ to 0.75 g/l.^[39a]^ However, fatty alcohols naturally synthesized in many (micro)organisms such as *E. coli* are limited to even‐chain lengths due to the selectivity of the FA synthases for two‐carbon building blocks.^[40]^ Cao *et al*. found that odd‐chain fatty alcohols can be produced in *E. coli* by expressing *Os*DOX.^[19]^ By overexpressing an endogenous fatty ACP thioesterase (TE) and aldehyde reductase with *Os*DOX, a titer of 1.95 g/l (or 0.105 g/l*h) of odd‐chain fatty alcohols was achieved. Tridecanol was obtained as the major product with 66 % content; undecanol and pentadecanol were also detected attributing 19 % and 15 % in the product mixture, respectively. These odd‐chain fatty alcohols are used as flavoring ingredients or can be further processed into nonionic ethoxylate surfactants such as polyoxyethylene tridecyl ether. Based on this microbial system, the production of odd‐chain wax esters starting from fatty alcohols was already suggested as a future application.^[39b]^ Furthermore, the production of even‐chain alkanes from odd‐chain FALs by the tandem activities of *Os*DOX and a cyanobacterial aldehyde deformylating oxygenase (cADOs) from *Synechococcus elongatus* PCC 7942, which is also called aldehyde decarbonylase (AD), was demonstrated.[^19^, ^41^] Based on this system, Foo *et al*. detected up to 0.3 mg/l C_12_, C_14_ and C_16_ alkanes in response to feeding FFAs to a *S. cerevisiae* culture, as well as low titers of 0.03 to 0.04 mg/l of C_14_ and C_16_ alkanes from *de novo* synthesis,^[41]^ while Cao *et al*. achieved up to 5.2 mg/l of a C_12_ and C_14_ alkane mixture in fermentations of *E. coli* using glycerol as the main carbon source.^[19]^ Despite the low yields, these studies introduced α‐DOXs for the production of even‐chain alkanes in a microbial system for the first time.

So far, most of α‐DOX studies have focused on FAs as the (natural) substrates.[^20^, ^42^] Most recently, *Os*DOX was also found to convert l‐pipecolic acid to δ‐valerolactam, emphasizing that the potential substrate range is not limited to FAs.^[43]^ Investigation of non‐FA substrates and potential protein engineering endeavors will certainly provide access to new α‐DOX‐based products.

### 3.4 Measuring α‐DOX activity

The ever‐expanding number of new biocatalysts from natural resources and from protein engineering studies ‐ not only for the synthesis of FALs and related products ‐ calls for tools for their rapid characterization and the reliable detection and quantification of target compounds.[^24a^, ^44^] In the following, analytical methods to determine α‐DOX activity are divided into (1) direct methods determining the target FAL products including chromatographic analysis and (2) indirect methods based on measuring the consumption of oxygen, which is the co‐substrate of α‐DOXs. A selection of analytical procedures for aldehydes produced by biocatalysis was also recently reviewed by Kazimírová and Rebroš.^[18c]^

Briefly, gas chromatography (GC) equipped with mass spectrometer (MS) or a flame ionization detector (FID) is the most direct tool routinely used for the detection and quantification of α‐DOX products.[^2^, ^15b^, ^18a^, ^32b^] However, like most chromatographic methods, sample preparation can be tedious and time‐consuming due to the extraction of compounds from aqueous reaction solutions and/or their derivatization to increase their volatility for subsequent GC analysis. Furthermore, short FAs or FALs, which are highly volatile, could evaporate during the sample preparation, leading to the loss of these compounds. This might result in underestimation of their concentrations. Another drawback of chromatographic methods is the low to moderate sample throughput.[^24a^, ^45^] Besides GC, FALs produced by α‐DOXs can be measured spectrophotometrically, for example, using 2,4‐dinitrophenylhydrazine (2,4‐DNPH).^[18a]^ Here, aldehydes react with 2,4‐DNPH to the 2,4‐dinitrophenylhydrazone derivative, which is then detected spectrophotometrically. However, the 2,4‐DNPH assay requires multiple steps requiring organic solvents to recover the insoluble 2,4‐dinitrophenylhydrazone derivatives. Complementary, Ressmann *et al*. developed a high‐throughput (HT) assay to quantify heterologously produced aldehydes in living cells.^[46]^ Exogenously added 2‐amino‐benzamidoximes (ABAOs) and target aldehydes form dihydroquinazolines, which can be detected spectrophotometrically.^[47]^

**Figure 4:**
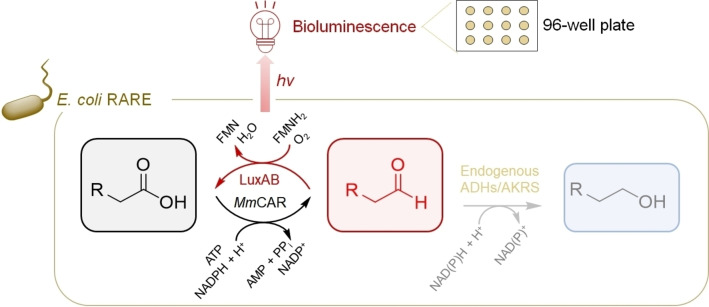
LuxAB‐based HT detection of aldehydes *in vivo*. The co‐expression of *Mm*CAR and the accessory *Ni*PPT (not shown for clarity) with the biosensor LuxAB provided a HT assay for the sensing of aldehydes.^[15c]^ These are produced enzymatically from carboxylic acid substrates in *E. coli* RARE. Subsequently, LuxAB oxidizes aldehydes to the corresponding carboxylates, emitting bioluminescence. Endogenous ADHs and AKRs can reduce intermediate aldehydes to the corresponding alcohols.

Instead of the detection and quantification of reaction products from aldehydes and chemical auxiliaries, luciferase assays have been employed for the direct detection of aldehydes.[^15c^, ^18a^] These are based on measuring bioluminescence emitted during the oxidation of (long‐chain) FALs to the corresponding FAs. The reaction is catalyzed by bacterial luciferases in the presence of reduced flavin mononucleotide (FMNH_2_) and O_2_ (Figure 4).[^15c^, ^18a^, ^48^] Kaehne *et al*. employed a bacterial luciferase (LuxAB from *Vibrio harvey*) to assess α‐DOX activity. In this study, *Os*DOX produced FALs, detected in crude cell extract from *E. coli*, by adding the purified luciferase and supplying FMN and sodium dithionite as reducing agent.^[18a]^ Generally, luciferases are excellent photoemitters in terms of quantum yield, defined as the ratio of the number of emitted photons to that of absorbed photons, hence, the detection of bioluminescence is highly sensitive.^[49]^ This method shows very low background signals, which contributes to high reproducibility, too. Importantly, it is possible to perform this assay in 96‐well microtiter plates, offering higher sample throughput. Along this line of research, Bayer *et al*. introduced LuxAB from *Photorhabdus luminescens* into the engineered *E. coli* RARE strain, exhibiting reduced aldehyde reduction (Figure 4).[^15c^, ^36b^] Their whole‐cell set‐up could not only directly detect reported FALs (*e. g*., C_8_ to C_12_) and new substrate aldehydes including monoterpenes and aromatic aldehydes in a 96‐well plate format; the production of aldehydes could be monitored *in situ* through the co‐expression of FAL‐producing enzymes like the ADH AlkJ, a choline oxidase variant from *Arthrobacter chlorophenolicus* (*Ac*CO‐6),^[17b]^ and *Mm*CAR. Noteworthy, FAL intermediates synthesized by FARs could be also monitored by the integration of the LuxAB gene into the genome of *A. baylyi* from *P. luminescens* previously.[^14a^, ^15c^, ^21a^] In spite of all these merits, the LuxAB‐based HT assay has some limitations. For example, the luminescent signals for different substrates are dependent on both the acceptances by the enzyme of interest for FAs and the LuxAB for FALs.^[50]^ Consequently, aldehydes that are poor substrates for LuxAB but are readily produced by a target enzyme will not be detected. Contrary, a poor substrate for the enzyme of interest yielding minimal amounts of aldehyde that is well‐accepted by LuxAB might show high bioluminescence. As a result, a deceptive substrate scope might be suggested.[^15c^, ^51^] Further, under HT screening conditions, both the maximal bioluminescence signal and the background bioluminescence depend on the heterologously expressed oxidoreductases ‐ AlkJ, *Ac*CO‐6, and *Mm*CAR. This divergence is not only based on the substrate preferences of the target oxidoreductases but could be explained by their distinct enzymatic features including the acceptance of intracellular FAs as substrates by *Mm*CAR, increasing the background bioluminescence, or the production of H_2_O_2_ by *Ac*CO‐6, decreasing cell viability. Although the introduction of an experimental cut‐off value for bioluminescence signals was sufficient to address varying backgrounds and allowed the confirmation of the substrate scopes of different oxidoreductases, these factors need to be taken into account, if the LuxAB system is tested with α‐DOXs in the future. Since Bayer *et al*. could already report the detection of products of α‐DOX‐catalyzed reactions (*e. g*., 12‐methyltridecanal) *in vivo*,^[15c]^ this system is of significant interest to identify novel α‐DOX enzymes and pre‐assess substrate scopes in HT, although absolute quantification of aldehydes might be difficult due to the transient nature of bioluminescence signals and their dependence on reaction conditions.^[52]^

All the direct measurements highlighted above require ‐ if applicable ‐ calibration curves for each compound to be quantified. Alternatively, oxygen depletion assays using oxygen sensors can be used to indirectly measure the conversion of FAs by α‐DOXs since the reaction consumes O_2_ as the co‐substrate.[^2^, ^18b^, ^32a^] *In vitro*, substrate spectra of α‐DOXs can be quantitatively investigated as O_2_ consumption occurs as long as the substrate is accepted.[^2^, ^18b^, ^32a^] By determining the amount of dissolved oxygen consumed by the α‐DOX enzyme during a specified time, the enzymatic specific activity can be determined for a wide range of substrates. However, in order to precisely examine the actual amount of oxygen actually consumed by the enzyme, the oxygen ingress from the atmosphere must be prevented. Moreover, this indirect approach measures the consumption of the co‐substrate but not the formation of the target FAL product. This is a drawback, especially for α‐DOXs generating hydroxyl FAs as side‐products.[^32b^, ^37^]

This selection of methods is by far not exhaustive but emphasizes that, based on current literature precedence, a combination of different analytical methods is highly recommended to reliably determine α‐DOX activities.

### 3.5 Key considerations for functional expression of α‐DOXs and biocatalytic applications

Whereas the importance of heme for the catalysis of α‐DOXs was already introduced above, its role as bottleneck during the heterologous production of (functional) α‐DOXs has not been fully addressed yet.^[28]^ We expressed various α‐DOXs in *E. coli* but yields of (active) enzyme varied highly from batch to batch. This could be partly attributed to incomplete heme incorporation,^[18b]^ which is essential for activity. Low heme occupancy can be due to insufficient intracellular amounts of the cofactor (or its precursors) in (heterologous) hosts and/or the intrinsic heme‐binding properties of α‐DOXs. These issues can be solved by expressing the enzymes in a different host or enhancing heme synthesis or by protein engineering, respectively.^[24a]^ The *R_z_* value, representing the normalized heme content by the amount of enzyme, is an important parameter.^[28]^ Based on literature and our study, the *R_z_* value of *Os*DOX appears to be higher than of cyanobacterial homologues.[^18b^, ^28^] The sequence and structural comparisons made by us indicated a high variation among α‐DOXs in the residues expected to associate with the heme cofactor.[^18b^, ^34b^] In this regard, heme‐coordinating residues of cyanobacterial (and other non‐plant) α‐DOXs can be engineered in a manner mimicking the corresponding amino acids in plant α‐DOXs. This will be meaningful if cyanobacterial α‐DOXs can be engineered to have a higher and consistent heme occupancy because ‐ to date ‐ only cyanobacterial α‐DOXs seem to have a preference for shorter chain FAs as demonstrated earlier.

A prerequisite for the heme incorporation is that host cells provide sufficient amounts of this cofactor, especially in recombinant hosts overexpressing a target hemeprotein, or that the heme is supplemented exogenously and imported sufficiently inside the cell. Since commonly used *E. coli* strains are not able to take up heme from the environment,^[53]^ the design of a heme‐protein expression system, which is based on the expression of the heme receptor ChuA circumvented this drawback.^[54]^ ChuA simply facilitates the uptake of heme from the culture medium.^[55]^ However, the use of additional plasmids increases the metabolic burden, consequently, this can for example interfere with the growth of host cells and lower protein yields.[^24a^, ^24b^] This was addressed by Fiege *et al*. who used the non‐pathogenic *E. coli* strain Nissle 1917, which possesses a chromosomal copy of the *chuA* gene, for the production of heme‐containing proteins.^[56]^

Alternatively, heme‐biosynthetic pathways have been engineered to increase cofactor availability in living cells. The first committed precursor towards heme production is 5‐aminolevulinate (ALA) in *E. coli* and other organisms. ALA can be synthesized from l‐glutamate or from glycine and succinyl‐CoA. Both pathways are tightly regulated at different points in the metabolic pathways, for example, by feedback regulation through intermediates and the final heme product.^[57]^ The optimization of heme production features the plasmid‐based and genomic overexpression of different heme‐biosynthetic enzymes including ALA synthases (*e. g*., *hemA* from *Rhodobacter sphaeroides*) and pantothenate kinase (*coaA* from *E. coli*) or the soluble cytochrome b5 from rat. The latter increased the intracellular heme concentration, even though the cytochrome does not participate directly in normal cellular regulation.^[57a]^ Motivated by the many applications in healthcare and food industries, the group of Lee engineered *E. coli* for the secretory production of heme. Their study targeted both the glutamate route towards ALA and the downstream pathway for heme biosynthesis.^[58]^ Metabolic engineering also included the knock‐out of putative heme‐degrading enzyme activities (encoded by the *ldhA*, *pta*, and *yfeX* genes in the *E. coli* genome) and resulted in >7.5 mg/l of total heme of which only 1.25 mg/l were actually detected in the cultivation medium. Optimization of fed‐batch fermentations based on glucose as the carbon source supplemented with l‐glutamate reached >230 mg/l of total heme produced, of which about 150 mg/l were exported when co‐expressing the CcmABC heme exporter.[^58^, ^59^] The lessons from this study can be certainly transferred and adjusted to *E. coli* and other recombinant strains for the expression of α‐DOXs.

Independent from the cofactor availability, the high insolubility of α‐DOX enzymes derived from membrane association is another obstacle^[34a]^ and might be overcome by expressing them in alternative biotechnological hosts like yeasts (*e. g*., *Pichia pastoris* and *S. cerevisiae*). Yeasts possess membrane‐surrounded organelles, which provide a higher binding capacity for membrane proteins, and have been successfully employed for the high‐yielding production of difficult‐to‐express proteins.^[60]^

Lastly, the lipophilic nature of FA substrates (*i. e*., their low solubility in aqueous media) is inevitable and can be target by the engineering of reaction and process conditions. Closely related, but to be addressed by genetic and metabolic engineering, is the uptake of FAs by microbial cells.^[61]^ Unspecific transporters ‐ such as AlkL for hydrophobic compounds (including FA precursors) and FA‐specific transporters like FadL ‐ have been introduced to enhance the uptake of these molecules.[^14b^, ^61^, ^62^] Some of these studies but also our unpublished preliminary data using FadL‐overexpressed in recombinant *E. coli* cells suggested that increased intracellular amounts of FFAs were cytotoxic, adversely decreasing product yields. More practically, detergents or cosolvents have been supplemented to improve the FA solubility and/or assist cell permeabilization in α‐DOX‐mediated microbial reactions.[^15b^, ^18a^] Cosolvents can also act as substrate reservoir, increasing substrate loads and decreasing substrate toxicity.[^15e^, ^63^] However, in all these cases, detailed optimization needs to be conducted to meet the physicochemical properties of the compound to be manufactured and, equally important, industrial process parameters.[^15d^, ^64^] When it comes to cosolvents, for example, a solvent that is readily removable (*i. e*. has a low boiling point) from the product in the downstream processing needs to be chosen. As a strategy to facilitate this optimization, we suggest a biosensor‐based high‐throughput approach instead of the routinely used GC since GC‐based approaches are tedious and labor‐intensive, and even impossible when for instance, a detergent highly interferes with the extraction of hydrophobic compounds from aqueous solution.

## 4 Conclusions

Heme‐dependent α‐DOXs are emerging biocatalysts and complement the toolkit for the bio‐based production of industrially relevant flavor and fragrance compounds, amongst others. The recently discovered phylogenetical diversity of the α‐DOX family and the first characterization of cyanobacterial α‐DOXs not only expanded the sources of novel α‐DOX beyond the well‐described enzymes from plants; the substrate specificity of the characterized cyanobacterial α‐DOXs is different and advances the biocatalytic production and tailoring of FA‐derived, natural and non‐natural chemicals. These findings, substantiated by sequence alignments performed by our group, provide potential starting points for (semi‐)rational protein engineering, targeting amino acid residues to alter substrate scopes or improve overall performance by stabilizing heme incorporation in recombinantly produced α‐DOXs. Further, this review highlighted strategies aiming at the improved (functional) expression of α‐DOXs and factors influencing process conditions including the supplementation of detergents. So far, only a few α‐DOX enzymes have been systematically characterized. To facilitate this, selected analytical methods were presented, of which biosensors like bacterial luciferase‐based systems for the rapid detection of aldehydes ‐ FALs in particular ‐ in living cells point towards the HT screening of novel α‐DOX candidates. Taken all this into account, the prospects for α‐DOXs for biocatalytic and industrial applications never looked brighter.

## Conflict of interest

The authors declare no conflict of interest.

## Biographical Information

*In Jung Kim received her Ph.D. in Food Bioscience and Technology from the University of Korea (South Korea) under the supervision of Prof. Kyoung Heon Kim in 2014. Subsequently, she performed her postdoctoral research in Prof. Kim's laboratory. In 2020, she joined the Bornscheuer group at the University of Greifswald (Germany) as a postdoctoral scientist on a project related to enzymatic synthesis of flavor compounds. Currently, she performs research at the University of Korea*.

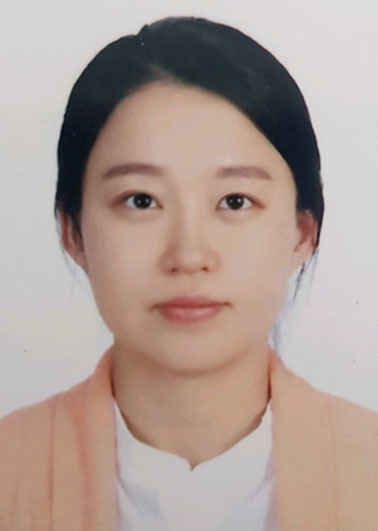

## Biographical Information

*Henrik Terholsen is part of the strategic network funding program ComBioCat of the Leibniz Association. He received his M.Sc. in the Industrial Organic Chemistry and Biotechnology group at Bielefeld University, and is currently pursuing his Ph.D. in the Bornscheuer group. His main research interests are protein engineering, artificial metalloproteins, biocatalysis and sustainable chemistry*.

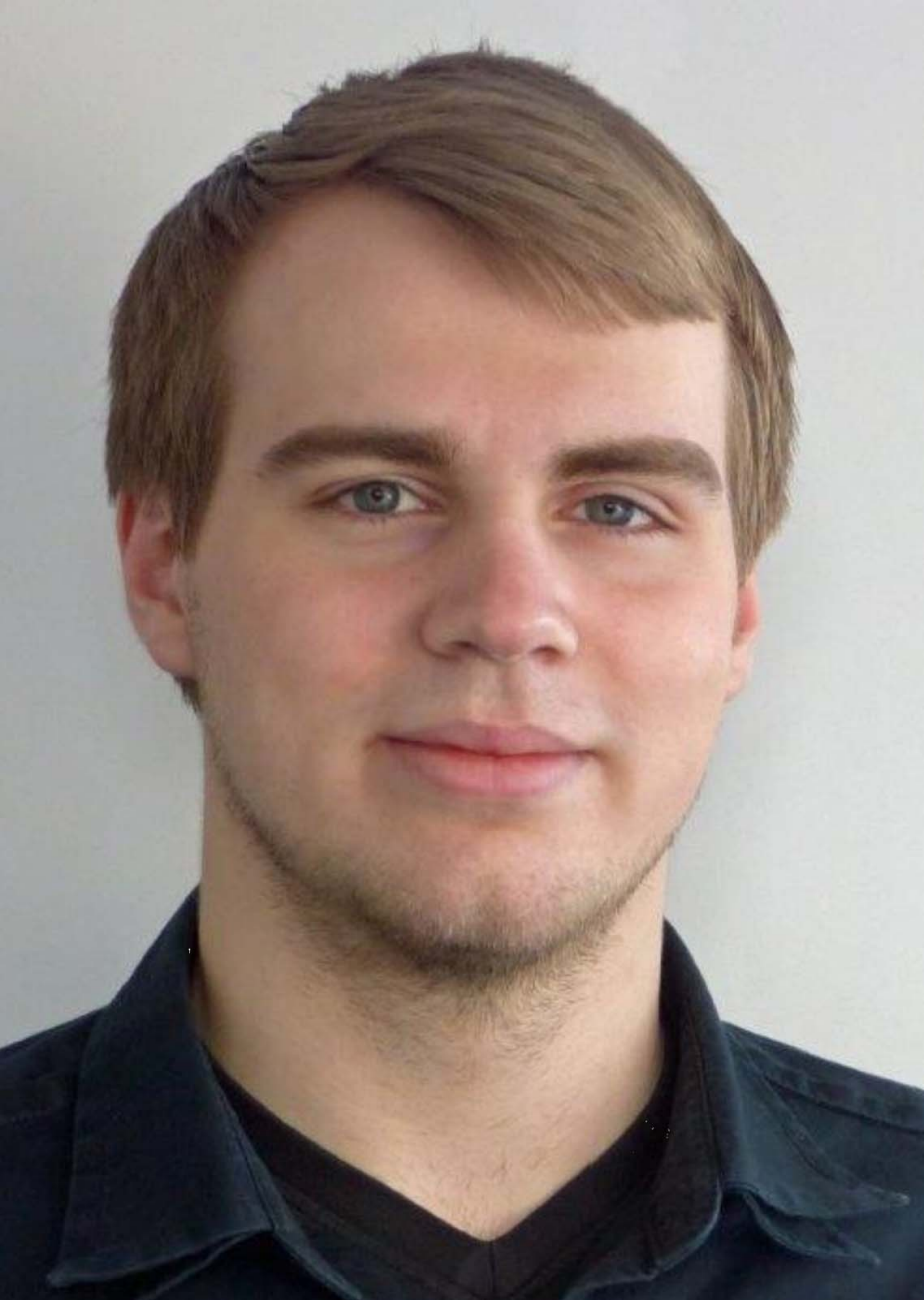

## Biographical Information

*Thomas Bayer graduated in the Molecular Biology program from the University of Vienna and received his Ph.D. in Bioorganic Chemistry at the TU Wien (Austria) before joining the Bornscheuer group as a postdoctoral fellow. He received the Erwin Schrödinger fellowship by the Austrian Science Fund (FWF), which included scientific stays at the University of Greifswald (Germany), the Albert Einstein College of Medicine in New York City (USA) in 2019–2020, and at the Institute of Molecular Biotechnology at the TU Graz (Austria). He has been back with the Bornscheuer group since February 2022*.

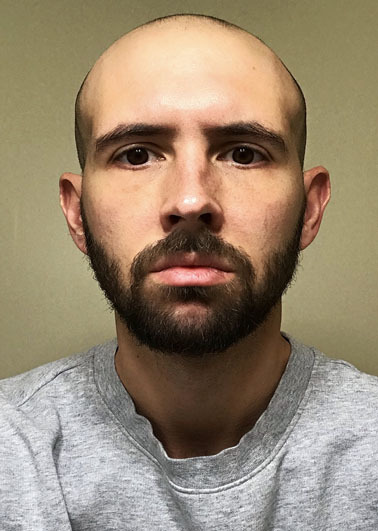

## Biographical Information

*Uwe T. Bornscheuer studied chemistry and received his Ph.D. in 1993 at Hannover University followed by postdoctoral research at Nagoya University (Japan). In 1998, he completed his Habilitation at Stuttgart University on the use of lipases and esterases in organic synthesis. He has been Professor at the Institute of Biochemistry at Greifswald University since 1999. Among other honors he was awarded the BioCat2008 Award. He was recently recognized as ‘Chemistry Europe Fellow’. His current research interests are on the discovery and engineering of enzymes from various classes for applications in organic synthesis, for flavors and fragrances, in lipid modification, and the degradation of plastics or complex marine polysaccharides*.

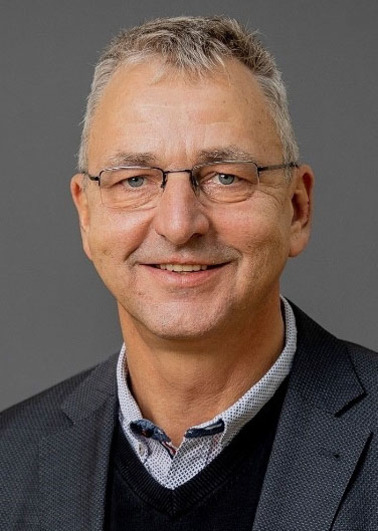
